# GSDMD is a novel predictive biomarker for immunotherapy response: in the pan-cancer and single cell landscapes

**DOI:** 10.3389/fimmu.2025.1570901

**Published:** 2025-05-26

**Authors:** Li Juan Huang, Feng Chen, Lin Chen, Shi Tong Zhan, Ming Min Liu, Jiang Dong Xiang, Qin Yi Zhang, Ye Yang

**Affiliations:** Obstetrics and Gynecology Department, Shanghai General Hospital, Shanghai Jiao Tong University School of Medicine, Shanghai, China

**Keywords:** pan-cancer, pyroptosis, GSDMD, immunotherapy, prognosis

## Abstract

**Background:**

Gasdermin D (GSDMD), a key executor of pyroptosis, has been implicated in modulating the tumor immune microenvironment. However, its role as a predictive biomarker for immunotherapy response remains unclear.

**Methods:**

We conducted a pan-cancer analysis of GSDMD expression across TCGA datasets and investigated its association with tumor mutational burden (TMB), microsatellite instability (MSI), and mismatch repair (MMR) status. Immunological relevance was further assessed by correlating GSDMD expression with immune cell infiltration and immune checkpoint gene signatures. We performed single-cell RNA sequencing analysis to investigate the immune cell populations and immunological pathways associated with GSDMD expression. Finally, organoid-based functional assays confirmed that Poly ADP-ribose polymerase inhibitors (PARPi) exert antitumor effects at least in part by enhancing GSDMD-mediated pyroptosis.

**Results:**

GSDMD was found to be aberrantly expressed in multiple tumor types and positively correlated with TMB, MSI, and immune checkpoint expression. High GSDMD expression was associated with increased infiltration of pro-inflammatory immune cells. In organoid models, GSDMD expression influenced sensitivity to PARPi, suggesting a potential role in shaping the immune-responsive phenotype.

**Conclusion:**

Our findings highlight GSDMD as a potential biomarker for predicting immunotherapy response and as a modulator of tumor-immune interactions. These results provide a foundation for future studies exploring GSDMD-targeted strategies to enhance immunotherapeutic efficacy.

## Introduction

Cancer treatment has long been complicated by tumor heterogeneity, treatment resistance, and the persistent challenges of recurrence and metastasis (1, 2). Traditional therapeutic approaches often fail to adequately address these complexities, underscoring the urgent need to identify new treatment targets and strategies to enhance the effectiveness of cancer therapies (3). Significant obstacles in cancer treatment include the difficulty in identifying specific tumor markers, the scarcity of targeted therapies, and the limited specificity and efficacy of conventional radiotherapy and chemotherapy (4).

In this context, pyroptosis—a form of programmed cell death mediated by the Gasdermin (GSDM) family, particularly Gasdermin D (GSDMD)—has garnered considerable attention (5, 6). GSDMD plays a pivotal role in immune responses and is closely linked to immune cell activity within the tumor microenvironment, positioning it as a promising target for improving cancer therapy. In recent years, our research team has pioneered investigations into pyroptotic mechanisms within endometrial cancer (EC) pathogenesis. Our prior investigations have established that key pyroptosis-associated proteins - NLRP3 inflammasome components, caspase-1, and GSDMD - exhibit significant overexpression in both endometrial carcinoma tissues and cellular models. Through subcutaneous xenograft experiments, we demonstrated that GSDMD-mediated pyroptosis exerts tumor-suppressive effects through growth inhibition (7).

Given the widespread occurrence of pyroptosis across various cancers, a pan-cancer approach is crucial for understanding the variations in GSDMD expression, distribution, and mutations across different tumor types and organs. These differences could have profound implications for both broad-spectrum and tumor-specific therapeutic strategies. Moreover, GSDMD’s high expression in immune-related organs naturally leads to exploring its role in immune modulation and its potential impact on immunotherapy. GSDMD is poised to play multifaceted roles in cancer, encompassing the regulation of cell pyroptosis and immune modulation within the tumor microenvironment (7–9). By investigating the biological functions and regulatory mechanisms of GSDMD in various cancers, this study aims to enhance our understanding of its involvement in tumorigenesis, progression, and treatment response. Ultimately, this research could pave the way for innovative cancer therapies that integrate both pyroptosis and immune modulation.

## Materials and methods

### Identification of GSDMD expression and survival analysis based on bioinformatics databases

We evaluated GSDMD expression in tumor tissues compared to normal tissues using the TIMER2.0 (http://timer.cistrome.org/, accessed on January 4th, 2024) and GEPIA2 (http://gepia2.cancer-pku.cn/, accessed on January 4th, 2024) databases (10), both of which are based on the TCGA database, encompassing 33 tumor types. In GEPIA2, a p-value cutoff of 0.05 and a log2(fold change) cutoff of 1 were applied. Additionally, we supplemented this analysis by downloading RNA-seq data for 33 types of tumor and normal tissues from the TCGA database (https://portal.gdc.cancer.gov/, accessed on January 6th, 2024) and the GTEX database (https://gtexportal.org, accessed on January 5th, 2024). To eliminate batch effects arising from different data sources, the ComBat method from the sva package was employed for batch correction. The ComBat function was used to standardize and adjust the expression data, resulting in a batch-effect-corrected expression matrix. Principal component analysis (PCA) was used before and after batch effect removal to evaluate the effectiveness of the correction. Differential expression of GSDMD in tumor versus normal tissues was visualized using ggplot2 in R 4.3.3.

We used clinical data from the TCGA database to perform multivariate Cox proportional hazards regression to assess the association between GSDMD, age, and gender with survival outcomes. Benjamini-Hochberg FDR correction was applied to control for false positives. FDR values were calculated for each cancer type, and only results with FDR < 0.05 were considered significant.

The prognostic value of GSDMD, including Overall Survival (OS) and Progression-Free Survival (PFS), was analyzed across 33 tumor types using TCGA data in GEPIA2.

### GSDMD and biomarkers in cancer immunotherapy: TMB, MSI, and MMR

Tumor Mutational Burden (TMB) is defined as the number of base mutations per million tumor cells (11), and is recognized as a quantifiable biomarker of immune response, reflecting the mutation load within tumor cells (12). Microsatellite Instability (MSI), resulting from DNA mismatch repair deficiency (MMRd), is linked to patient prognosis (13). Both TMB and MSI are valuable in predicting the effectiveness of immunotherapy, with patients exhibiting high TMB or MSI-H generally showing better responses to immune checkpoint inhibitors, such as PD-1 or PD-L1 inhibitors. Mismatch Repair (MMR) is a genetic surveillance mechanism that detects and corrects mismatched nucleotides during DNA replication, thereby maintaining genetic stability (14).We downloaded TMB and MSI data of GSDMD from the TCGA database (15) and created a correlation heatmap to explore the relationship between GSDMD and MMR genes. We applied the Benjamini & Hochberg (BH) method to adjust for multiple comparisons. Adjusted p-values (FDR) <0.05 were used identify statistically significant correlations.

### GSDMD expression and immune cell infiltration

We evaluated the infiltration of 22 immune cell types across 33 tumor types using the CIBERSORT algorithm. The ESTIMATE score, defined as the sum of immune and stromal scores, serves as an indicator of the cellular immune microenvironment, often referred to as the “non-tumor score.” Immune-related genes play a crucial role in tumors (16, 17). participating in the regulation of immune responses within the tumor microenvironment. These genes influence immune evasion, immune surveillance of tumor cells, and the response to immune therapies, including immune checkpoint inhibitors. We utilized SangerBox (15) to analyze GSDMD’s modulation of immune checkpoints and immune-related genes. Tumor Immunotherapy Gene Expression Resource (18) (TIGER, http://tiger.canceromics.org/) is a comprehensive and publicly accessible web-based portal for integrative analysis of gene expression datasets related to tumor immunology. To investigate the relationship between GSDMD expression and survival after immunology treatment, we employed both bulk and single-cell transcriptomic datasets from the TIGER.

### Single-cell immune analysis

The GSE198550 dataset is a publicly accessible resource containing single-cell RNA-seq data of tumor-infiltrating immune cells from Gsdmdfl/fl Cx3cr1-cre and Cx3cr1-cre mice (19). B16F10 tumor cells were subcutaneously implanted into Gsdmdfl/fl Cx3cr1-cre and Cx3cr1-cre mice, and following PD-1 treatment, tumors were harvested for single-cell RNA-seq analysis. The analysis was conducted using Seurat v5 in R version 4.3.3, with a focus on immune cell profiling based on data from GEO accession GSE198550. Using the original data, cells were filtered based on the following criteria: nFeature_RNA > 300 & nFeature_RNA < 7,000, mitochondrial proportion < 10%, UMI counts per cell > 1,000, and exclusion of the top 3% largest cells. Additionally, cells with erythrocyte gene expression exceeding 3% of the total genes per cell were removed. Subsequently, log-normalization was applied, and batch effects were corrected using the Harmony package. After Harmony integration, we performed dimensionality reduction and clustering using the Seurat package. The top 50 dimensions were visualized by ElbowPlot to determine the optimal number of principal components. We used the first 10 Harmony components for neighbor finding (FindNeighbors) and tested multiple clustering resolutions (FindClusters) ranging from 0.1 to 1.0. For downstream analysis, we selected a resolution of 0.5, which identified 13 clusters in the KO group and 14 in WT. UMAP dimensionality reduction was applied to visualize clustering results. Various cell types were classified and annotated using the CellMarker 2.0 database (20). Visualization of the abundance of each cell type was achieved using ggplot2, and UMAP analysis was performed to map the distribution of Gsdmd across different cell types.

### The connection between immune cells: Monocle and CellChat

Pseudotime trajectory analysis was conducted using the Monocle2 package in R version 4.3.3. The cell dataset was initialized via newCellDataSet, and functions such as estimateSizeFactors and estimateDispersions were applied to normalize and model the data. Low-quality cells were filtered out using detectGenes with a minimum expression threshold set to 0.1.

In the inference and analysis of cell–cell communication, we utilized CellChat (21), a public database that includes ligands, receptors, cofactors, and their interactions, to discover new modes of cell–cell communication and construct cell–cell communication atlases.

### GSDMD-related enrichment analysis in cancer

For each cancer type in the TCGA dataset, patients were divided into high and low GSDMD expression groups based on the median expression level of GSDMD. Differentially expressed genes (DEGs) between the two groups were identified using the limma package in R. Genes with an adjusted p-value < 0.05 and |log2 fold change| > 1 were considered significantly differentially expressed.

Gene Set Enrichment Analysis (GSEA) was then performed on the ranked DEGs using the clusterProfiler (v4.8.1) and GSEA (v1.38.2) R packages. For cancer-related pathway analysis, the Hallmark gene set file (h.all.v7.1.symbols.gmt) was downloaded from the Molecular Signatures Database (MSigDB, https://www.gsea-msigdb.org/gsea/msigdb). Enrichment results were assessed based on the Normalized Enrichment Score (NES) and False Discovery Rate (FDR). Significantly enriched gene sets were defined by NES > 2 and FDR < 0.01. Significant pathways were visualized using bubble plots, with point size indicating the gene ratio and color representing the adjusted p-value.

### Patient-derived organoids culture and treatment

Fresh endometrial tumor tissues were collected and minced into ~1 mm³ pieces, followed by enzymatic digestion using collagenase IV (BioGenous, China) for 1 h at 37°C. The resulting cell suspension was filtered, centrifuged, and embedded in Matrigel droplets. Organoids were cultured in complete organoid medium (BioGenous, China). After 7–14 days of growth, organoids with similar diameters were randomly divided into treatment groups. PARP inhibitors (PARPi), including Niraparib (50 nM, Beyotime, China), Olaparib (200 nM, Beyotime, China), and Rucaparib (1 μM, Beyotime, China), were administered for 24 h. Pyroptosis was subsequently induced by stimulation with lipopolysaccharide (LPS, 50 ng/mL, 4 h, Beyotime, China) and Nigericin (10 μM, 30 min, Beyotime, China).

### Histological and morphological assessment

Organoids were fixed in 4% paraformaldehyde, embedded in paraffin, and sectioned at 4 µm thickness. Hematoxylin and eosin (H&E) staining was performed following standard protocols. Images were acquired using an inverted fluorescence microscope. Organoid diameters were quantified using ImageJ software, and at least five organoids were measured per condition.

### TUNEL assay

DNA fragmentation associated with pyroptotic or apoptotic cell death was assessed using a TUNEL staining kit (Bryotime, China) according to the manufacturer’s protocol. Organoid sections were counterstained with DAPI and imaged using a confocal fluorescence microscope.

### Western blotting

Organoids were harvested and lysed using RIPA buffer supplemented with protease and phosphatase inhibitors. Protein concentrations were determined using the BCA assay (Epizyme, China). Equal amoual amounts of protein (40 µg) were separated by SDS-PAGE and transferred onto PVDF membranes. Membranes were blocked in 5% non-fat milk and incubated overnight at 4°C with primary antibodies against GSDMD, CHMP4B, and GAPDH (loading control). HRP-conjugated secondary antibodies were applied, and signals were visualized using enhanced chemiluminescence (ECL, Millipore). Densitometry analysis of band intensities was performed using ImageJ. All data are presented as mean ± standard deviation (SD).

### Statistical analysis

Statistical significance in our study was determined using the Wilcoxon test, with significance levels annotated as follows: * for p < 0.05, ** for p < 0.01, and *** for p < 0.001. Analysis of variance (ANOVA) in GEPIA2 was employed to compare tumor samples with all normal samples. Spearman rank correlation coefficients were used to assess correlations between two groups. The Kaplan-Meier method was applied to evaluate the association between patient prognosis and GSDMD expression or mutation levels, with p < 0.05 considered statistically significant.

## Results

Expression landscape and prognostic value of GSDMD across human cancers.

**Figure 1:**
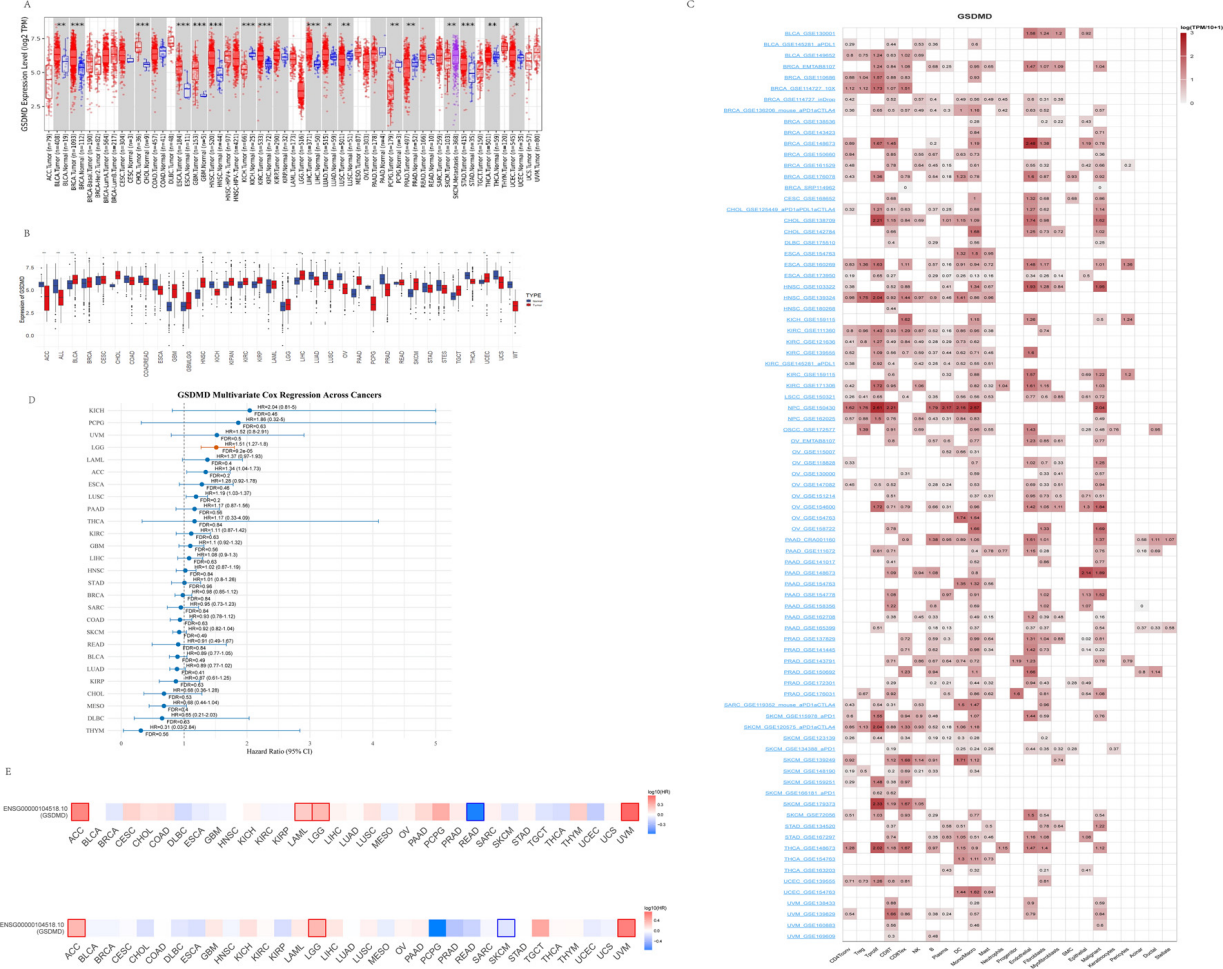
Expression pattern and survival analysis of GSDMD in normal and tumor tissues. **(A)** Expression of GSDMD in tumors compared to their corresponding adjacent non-cancerous tissues, sourced from the TIMER2 database. (mean ± SD; paired t-test). **(B)** Expression of GSDMD in tumors and normal tissues, sourced from the TCGA and GTEx databases. (mean ± SD; paired t-test). **(C)** Single-cell expression of GSDMD in different cell types, sourced from the TISCH database. **(D)** Forest plot of multivariate Cox regression analysis evaluating the prognostic significance of GSDMD expression across TCGA pan-cancer types. The analysis was adjusted for potential confounding factors, including age and gender. For each cancer type, the hazard ratio (HR), 95% confidence interval (CI), and false discovery rate (FDR) are presented. Cancers with significant prognostic association (FDR < 0.05) are highlighted. **(E)** Kaplan–Meier analysis of GSDMD expression in relation to overall survival (OS) and progression-free survival (PFS) across cancer types. Obtained from the GEPIA2 database. Significant results (p < 0.05) are marked by framed areas. *p < 0.05, **p < 0.01, ***p < 0.001.

We investigated the differential expression of GSDMD in tumor tissues compared to adjacent non-cancerous tissues across 15 tumor types (**Figure 1A**). GSDMD was significantly upregulated in Bladder Urothelial Carcinoma (BLCA), Breast Cancer (BRCA), and other tumors, while it was notably downregulated in Colon Adenocarcinoma (COAD), Kidney Chromosome (KICH), and several others. However, it is important to note that some results from the TIMER database lack corresponding non-cancerous tissue information. To address this limitation, we conducted further analyses using the TCGA and GTEx databases. Using R, we examined GSDMD expression across various tumors and their corresponding non-cancerous tissues, with outliers (beyond mean ± 6 standard deviations) removed (**Figure 1B**). Additionally, GSDMD was upregulated in Lower Grade Glioma (LGG) and Melanoma (SKCM), while showing downregulation in Uterine Carcinosarcoma (UCS).

We then explored the cellular distribution of GSDMD within the tumor microenvironment using the TISCH single-cell RNA sequencing database (**Figure 1C**). The relative expression levels of GSDMD across 33 cell types indicated widespread expression in various immune and malignant cells. After eliminating the interference of confounding variables including age and gender through multivariate Cox proportional hazards analysis, it was revealed that high GSDMD expression is significantly associated with an increased risk of LGG incidence (p < 0.001, HR = 1.51, 95% CI: 1.27–1.80)(**Figure 1D**). Furthermore, data from the GEPIA2 database showed a positive correlation between GSDMD expression and overall survival (OS) in SKCM, and a negative correlation in ACC, LGG, and Uveal Melanoma (UVM). When analyzing progression-free survival (PFS), GSDMD was positively correlated with Rectum adenocarcinoma (READ), and negatively correlated with ACC, LGG, and others (**Figure 1E**).

Collectively, these findings indicate that GSDMD exhibits cancer-type-specific expression patterns and is intricately linked to patient prognosis, suggesting a potential role in tumor development and progression.

### GSDMD and biomarkers in cancer immunotherapy: TMB, MSI, and MMR

**Figure 2:**
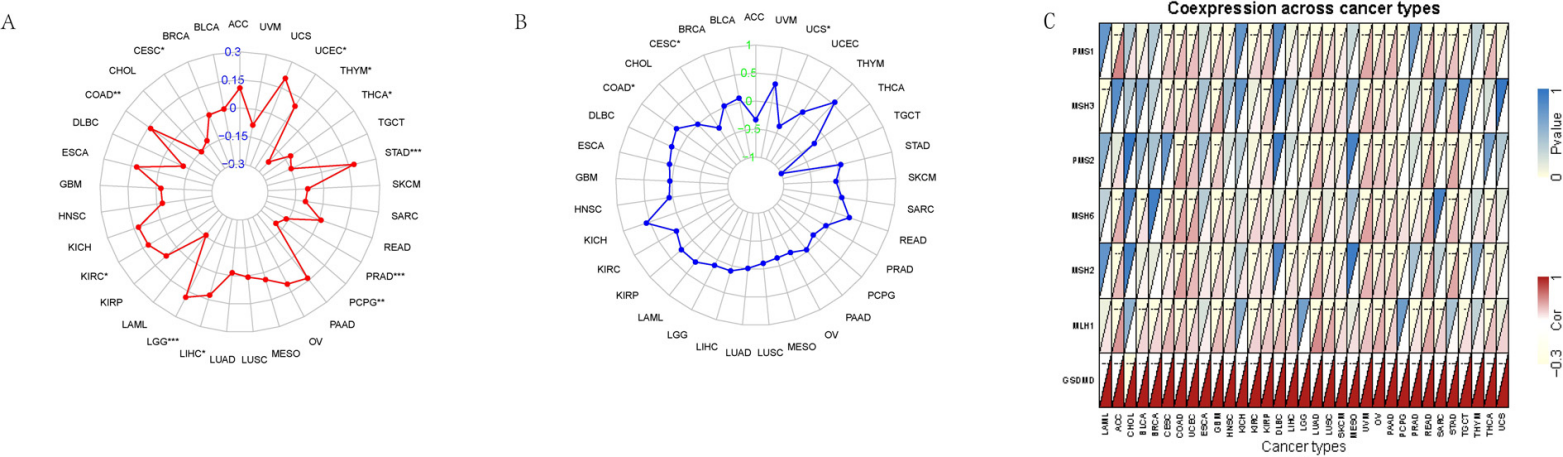
Association between GSDMD expression and genomic instability markers across TCGA pan-cancer types. **(A)** Spearman correlation between GSDMD expression and tumor mutational burden (TMB) across multiple cancer types. **(B)** Correlation between GSDMD expression and microsatellite instability (MSI) scores. **(C)** Correlation between GSDMD expression and the expression levels of key mismatch repair (MMR) genes. *p < 0.05, **p < 0.01, ***p < 0.001.

To further explore the immunological relevance of GSDMD in cancer, we investigated its association with three key biomarkers of immunotherapy response: tumor mutational burden (TMB), microsatellite instability (MSI), and mismatch repair (MMR) gene expression. Pearson correlation analysis revealed that GSDMD expression was significantly associated with TMB in several cancer types. Specifically, a positive correlation was observed in seven tumors, including Uterine Corpus Endometrial Carcinoma (UCEC) and Stomach Adenocarcinoma (STAD), while a negative correlation was found in five tumors such as Thymoma (THYM) and Thyroid Carcinoma (THCA) (**Figure 2A**). Similarly, analysis of MSI scores demonstrated a significant positive association with GSDMD expression in cancers including Colon Adenocarcinoma (COAD) (**Figure 2B**). Moreover, expression levels of MMR genes—PMS1, MSH3, PMS2, MSH6, MSH2, and MLH1—were positively correlated with GSDMD across most cancer types, with particularly strong associations noted in UCEC (**Figure 2C**).

To externally validate the prognostic relevance of GSDMD, data from the Tumor Immunotherapy Gene Expression Resource (TIGER) were utilized. In the SRA PRJNA482620 glioblastoma (GBM) cohort (22), which includes 66 patients treated with standard therapy and PD-1 inhibitors (nivolumab or pembrolizumab), higher GSDMD expression was associated with poorer overall survival (HR = 3.0719, p = 0.032, **Supplementary Figure 1**). Consistently, analysis of a second dataset involving 68 advanced melanoma patients treated with nivolumab (23), either post-ipilimumab or ipilimumab-naïve, demonstrated that high GSDMD expression predicted inferior survival outcomes (HR = 1.8781, p = 0.014, **Supplementary Figure 2**).

### Effect of GSDMD expression on immune cell infiltration in human cancers

**Figure 3:**
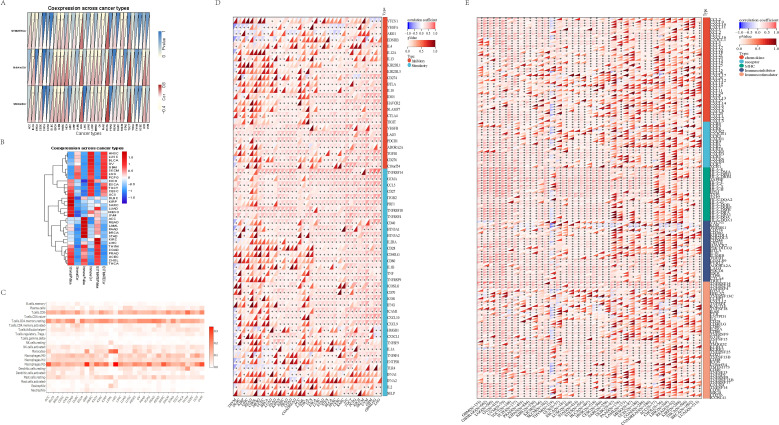
Tumor microenvironment and immune infiltration. **(A, B)** Immune score, stromal score, and immune microenvironment score (ESTIMATE score) across 33 tumor types in TCGA. Data are presented as box plots, with median values indicated. Statistical significance between tumor and normal tissues was assessed using the Wilcoxon rank-sum test. **(C)** Correlation between GSDMD expression and immune cell infiltration levels across pan-cancer types in TCGA. Immune cell infiltration levels were estimated using the CIBERSORT algorithm, and GSDMD expression was correlated with these levels using Spearman’s rank correlation. Data are presented as correlation coefficients (ρ) with corresponding p-values. **(D)** Relationship between GSDMD expression and immune checkpoint genes across various cancer types. **(E)** Correlation between GSDMD expression and immune-related genes in different cancers. The y-axis in the heatmaps corresponds to the names of the cancer types. *p < 0.05, **p < 0.01, **p < 0.001.

The tumor immune microenvironment plays a critical role in the initiation and progression of tumors. GSDMD expression shows differential correlations with immune, stromal, and tumor microenvironment (TME) scores across various cancers, revealing distinct patterns of immune cell infiltration and modulation of immune checkpoints and related genes. We calculated the immune score, stromal score, and ESTIMATE score for 33 tumor types and assessed the relationship between GSDMD expression levels and these scores. GSDMD expression was positively correlated with the immune, stromal, and TME scores in tumors such as OV and ESCA, while showing a negative correlation in UCEC (**Figures 3A, B**).

We also evaluated the infiltration of 22 immune cell types across 33 cancer types. GSDMD expression exhibited a strong correlation with monocytes, resting mast cells, resting dendritic cells, and activated CD4 memory T cells in most cancers (**Figure 3C**).

Using SangerBox (15), we analyzed GSDMD’s modulation of immune checkpoints and immune-related genes. In most tumors, GSDMD showed a negative correlation with immune checkpoint inhibitory genes such as V-set domain containing T cell activation inhibitor 1 (VTCN1), Vascular endothelial growth factor A (VEGFA), and Arginase 1 (ARG1), while it was positively correlated with immune checkpoint promoting genes including Tumor necrosis factor receptor superfamily member 14 (TNFRSF14), Perforin 1 (PRF1), Granzyme A (GZMA), C-C motif chemokine ligand 5 (CCL5), CD27, Integrin subunit beta 2 (ITGB2), Tumor necrosis factor receptor superfamily member 18 (TNFRSF18), and Tumor necrosis factor receptor superfamily member 4 (TNFRSF4) (**Figure 3D**). In various tumors, including GBM, LGG, and OV, GSDMD demonstrated a significant positive correlation with a majority of immune modulation genes, such as chemokine receptors, MHC, immune inhibitors, and immune stimulators (**Figure 3E**).

In the GSE135222 cohort, after anti-PD-1 treatment, the log2FC of GSDMD was 0.6526 in patients who responded to the immunotherapy compared to those who did not respond (p=0.019). This indicates that the expression level of the GSDMD gene was significantly higher in responders than in non-responders (**Supplementary Figure 3**). The RNA-seq data for Melanoma treated with anti-CTLA-4 therapy shows that the expression level of GSDMD significantly decreased after treatment (24) (log2FC = -1.1007), with a p-value of 0.021 (**Supplementary Figure 4**). To validate its clinical significance, transcriptomic data from immunotherapy cohorts were analyzed. In the GSE135222 dataset (25), which includes patients treated with anti-PD-1 therapy, GSDMD expression was significantly elevated in responders (log2FC = 0.6526, *p* = 0.019) compared to non-responders (**Supplementary Figure 3**).

Conversely, RNA-seq data from melanoma patients undergoing anti-CTLA-4 therapy revealed a significant downregulation of GSDMD post-treatment (log2FC = -1.1007, *p* = 0.021), suggesting dynamic modulation of GSDMD in response to distinct immune checkpoint inhibitors (**Supplementary Figure 4**).

### The relationship between GSDMD and immunity from a single-cell perspective

**Figure 4:**
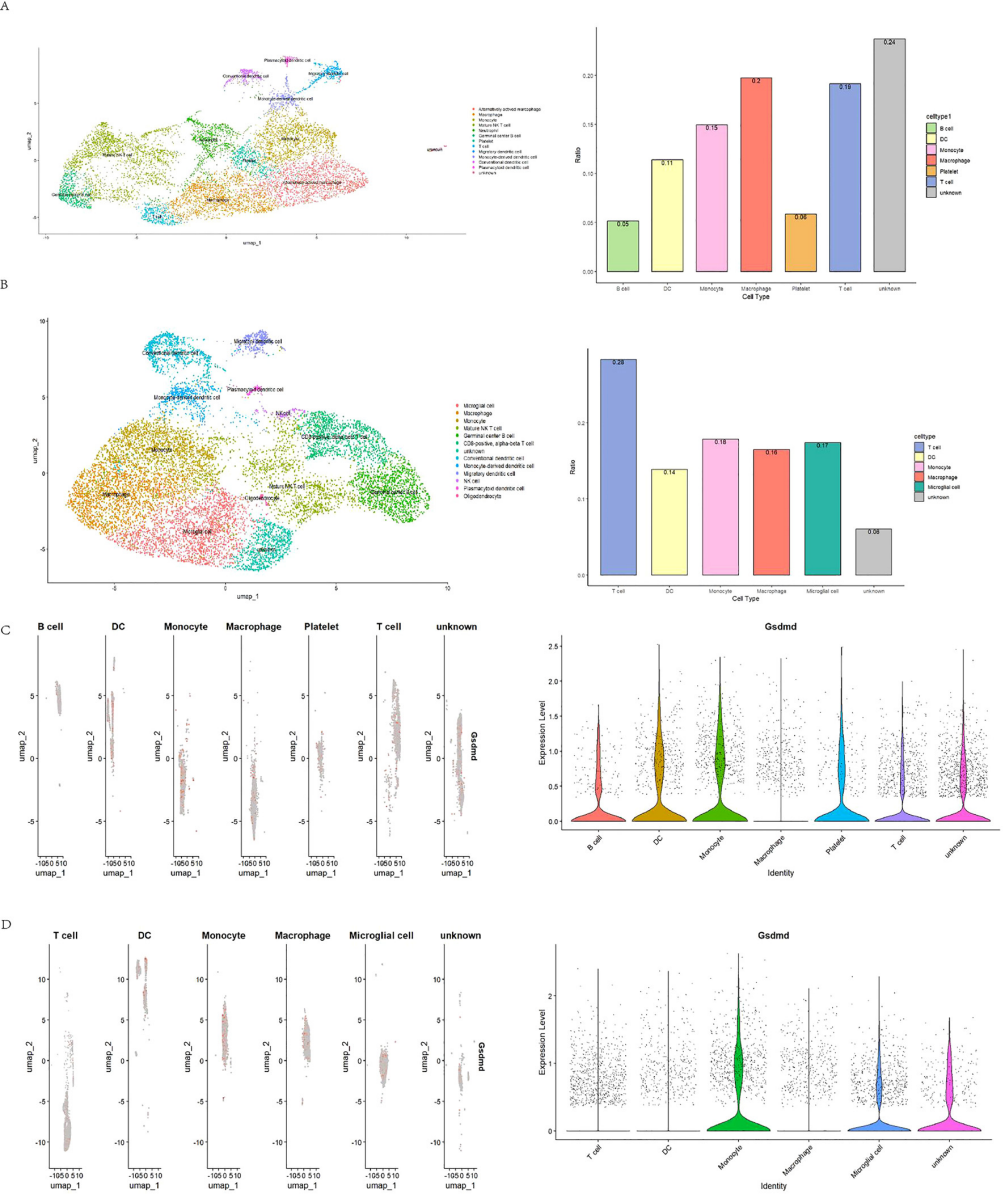
Visualization of annotated GSE198550 dataset. **(A)** UMAP plot of GSDMD-WT (wild-type) samples after annotation, showing cell type distribution across different clusters. The corresponding cell type proportion plot is presented. UMAP was generated using Seurat, with cell clusters annotated based on known marker genes. **(B)** UMAP plot of GSDMD-KO (knockout) samples after annotation, showing cell type distribution. The corresponding cell type proportion plot is also shown. The analysis was performed using Seurat and visualized with UMAP. **(C)** Gsdmd expression levels in different cell types within GSDMD-WT samples. Gsdmd expression was visualized using Violin plots, with expression levels compared across cell types. **(D)** Gsdmd expression levels in different cell types within GSDMD-KO samples. Violin plots depict expression levels.

We explored the correlation between GSDMD and immune responses at the single-cell level. Data from GSE198550, divided into GSDMD wild-type (WT) and GSDMD knockout (KO) groups, were analyzed, with cells from each group clustered into 14 distinct groups. Using the CellMarker2.0 database, the cell types within each cluster were identified based on their gene expression patterns. Dendritic cells (DC), monocytes, and T cells exhibited high expression in both groups. Our results also demonstrated that GSDMD knockout reduces the levels of B cells and macrophages while increasing the proportion of T cells (**Figures 4A, B**).

To further elucidate the relationship between GSDMD and immune cells, we compared the distribution of GSDMD across different cell types between the two groups. Notably, GSDMD was highly expressed in monocytes in both WT and KO mice. However, in GSDMD knockout mice, GSDMD expression was elevated in T cells and DCs, a pattern not observed in WT mice (**Figures 4C, D**).

### The connection between GSDMD-related immune cells

**Figure 5:**
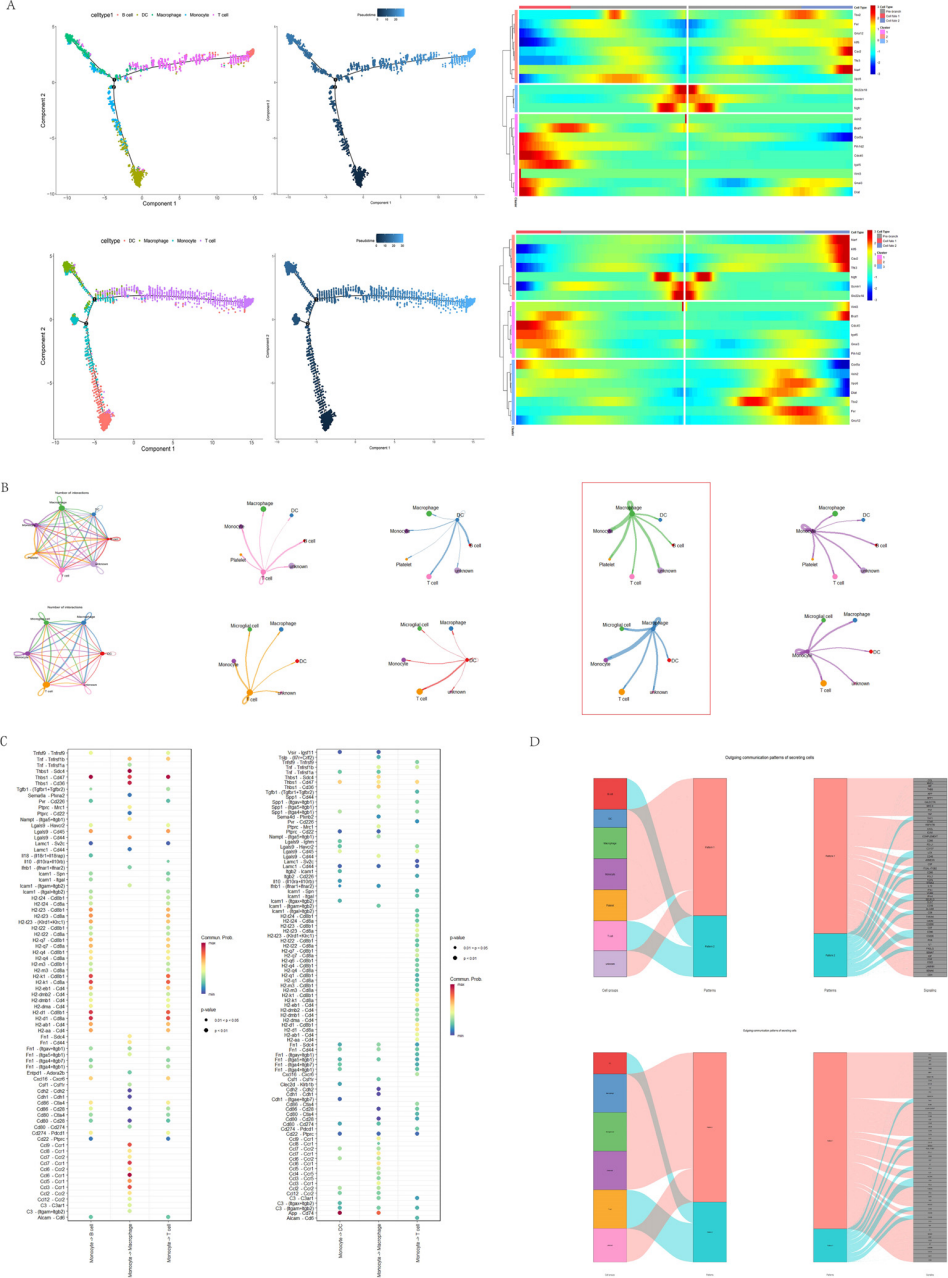
The connection between GSDMD-related immune cells. **(A)** Pseudotime developmental trajectories of two groups of cells (WT group: top; KO group: bottom), showing the progression of cellular states and associated gene expression profiles. Pseudotime analysis was performed using Monocle, and the trajectories were plotted to reflect developmental dynamics. **(B)** Strength of intercellular connections between different immune cell types in GSDMD-WT and GSDMD-KO groups. The connection strength was measured using cell-cell communication analysis and visualized in network plots. The analysis was conducted using CellPhoneDB, with the strength of interactions shown for both groups. **(C)** Signaling pathway weights between monocytes and other immune cells in the GSDMD-WT and GSDMD-KO groups. The pathway weights were calculated using ligand-receptor interaction analysis. The results are shown for the WT group on the right and the KO group on the left. **(D)** Signaling output of the two groups of cells, comparing the changes in signaling events between GSDMD-WT and GSDMD-KO samples.

We used the monocle2 package to compare the temporal dynamics of various cell types between the two groups. Our analysis primarily focused on the pseudotime trajectory of macrophages, monocytes, T cells, and dendritic cells (DCs), given the differences in cell subtypes. In both groups, the trajectory starts with DCs and culminates in T cells, exhibiting a similar developmental pattern. However, in the KO group, B cells are absent at the terminal stage, which may be associated with the reduced expression of genes such as Axin2, Xpo6, and Dlat at this stage (**Figure 5A**). To explore immune cell interactions, we employed the CellChat package. The signaling communication between subgroups is illustrated, with macrophages showing stronger connections with other subgroups in both groups (**Figure 5B**).

We focused on monocytes, where GSDMD is most highly expressed in both groups, to examine the differences in signaling pathways with other immune cells. In comparison to the WT group, the KO group exhibited a strong correlation with DCs and macrophages in the App-Cd47 pathway, which was nearly absent in the WT group. This suggests that GSDMD knockout may be related to the activation of this pathway (**Figure 5C**). The analysis of incoming and outgoing communication patterns in both the KO and WT groups revealed that the top five signaling pathways are “CCL,” “MHC-I,” “MIF,” “APP,” and “THBS” (with slight variations in order) (**Figure 5D**). Additionally, we analyzed intercellular signaling pathways separately in the WT and KO groups. In the WT group, the top three signaling pathways were CCL, MHC-I, and MIF, with CCL being the most prominent in monocytes. Monocytes in the WT group predominantly engaged in MHC-I and MIF pathways with B cells and T cells, while monocyte communication was primarily mediated through the CCL pathway (**Figure 5C**). In contrast, monocyte-DC connections in the KO group were relatively sparse and concentrated mainly in the CCL pathway (**Figure 5D**).

In conclusion, the dominant signaling pathways in both groups were CCL, MHC-I, MIF, APP, and THBS. In the WT group, macrophages exhibited stronger connections, particularly in the CCL, MHC-I, and MIF pathways, with CCL being prominent in monocytes. In the KO group, monocyte-DC connections were sparse and focused on the CCL pathway.

### Enrichment analysis of potential functions of GSDMD in cancers

**Figure 6:**
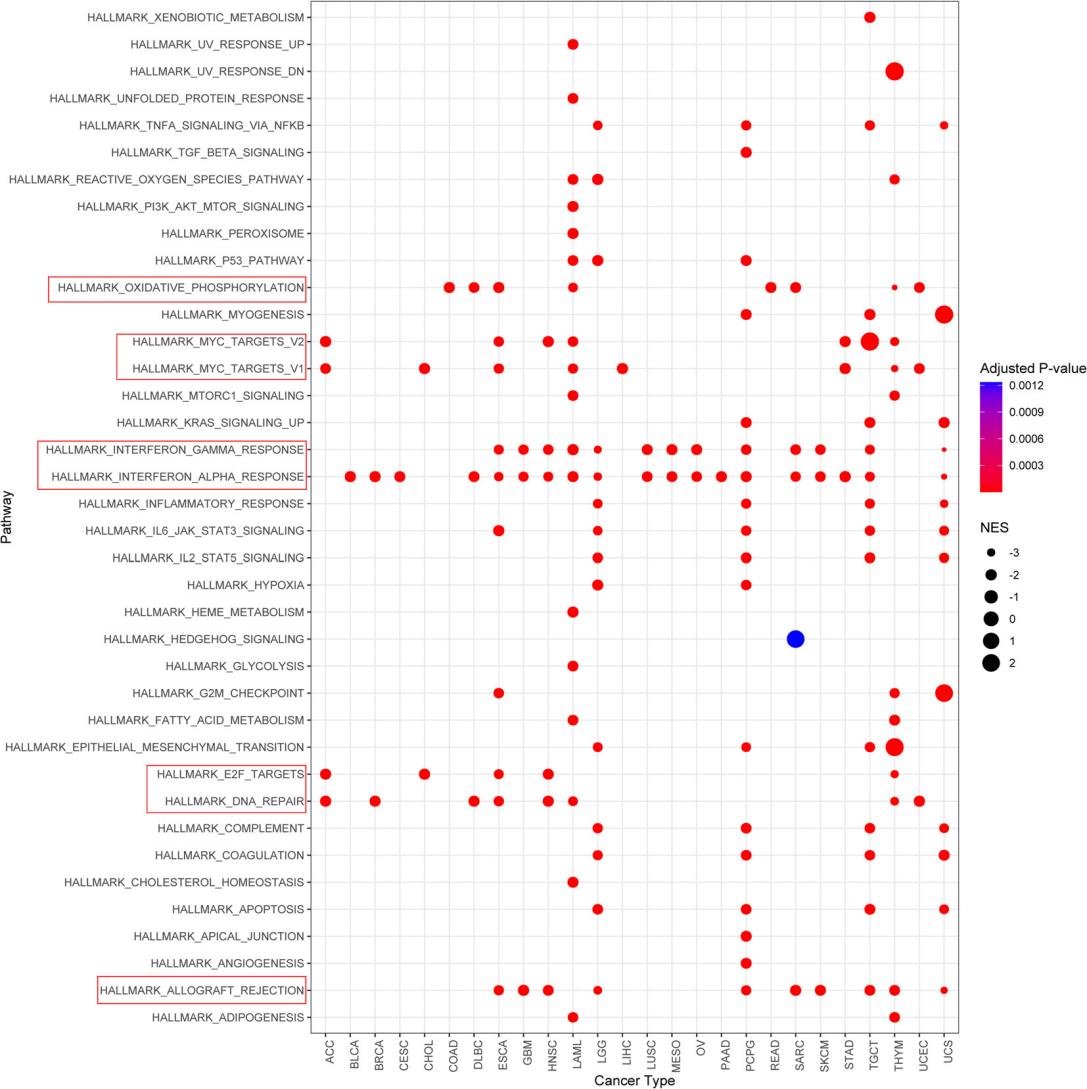
Gene set enrichment analysis identifies significantly enriched hallmark pathways. Pathways with normalized enrichment score (NES) > 2 and false discovery rate (FDR) < 0.01 are shown.

To investigate the potential functional roles of the input gene set, we performed pathway enrichment analysis using the MSigDB Hallmark gene sets. **Figure 6** illustrates pathways that were significantly enriched with a normalized enrichment score (NES) > 2 and a false discovery rate (FDR) < 0.01. The analysis revealed a significant enrichment of several immune-related pathways, including: interferon gamma response; interferon alpha response; allograft rejection. These pathways are strongly associated with immune activation, particularly T cell-mediated responses and Type I/II interferon signaling, suggesting that the gene set may be involved in shaping the tumor immune microenvironment.

In addition, enrichment was also observed in: oxidative phosphorylation; myc targets v1/v2; e2f targets; dna repair. Although not canonical immune pathways, these signatures are frequently associated with immune cell activation, metabolic reprogramming, and DNA damage responses that may influence tumor immunogenicity and responsiveness to immunotherapy.

Collectively, these findings suggest a potential link between the gene set and immune regulation, supporting further investigation into its role in modulating anti-tumor immunity.

### PARP inhibition enhances GSDMD-dependent pyroptosis in endometrial cancer organoids

Building upon our previous findings, we identified a novel regulatory axis involving endosomal sorting complex components Charged Multivesicular Body Protein 4B (CHMP4B) and vacuolar protein sorting 4 homolog A (VPS4A). These proteins were shown to counteract GSDMD-dependent pyroptosis through membrane remodeling mechanisms in EC models. Experimental modulation revealed that GSDMD silencing reduced multiple pyroptotic markers including propidium iodide-positive cell populations, calcium efflux, and IL-1β/LDH release. Conversely, CHMP4B/VPS4A depletion amplified these pyroptotic indicators. Complementary membrane integrity assays demonstrated that GSDMD inactivation decreased cellular perforations, while CHMP4B/VPS4A manipulation bidirectionally modulated membrane disruption patterns (26). To validate the functional role of GSDMD in pyroptosis, we established patient-derived organoids (PDOs) from a 57-year-old female diagnosed with FIGO 2023 stage IIC MMRd-type endometrioid endometrial carcinoma (ER/PR/MLH1/PMS2 negative; MSH2/MSH6 positive; partial p53 positivity; Ki-67 ~90%; germline BRCA1/2 wild-type). Organoids were treated with PARP inhibitors (PARPi), including niraparib, olaparib, and rucaparib, followed by stimulation with LPS and nigericin to induce pyroptosis.

**Figure 7:**
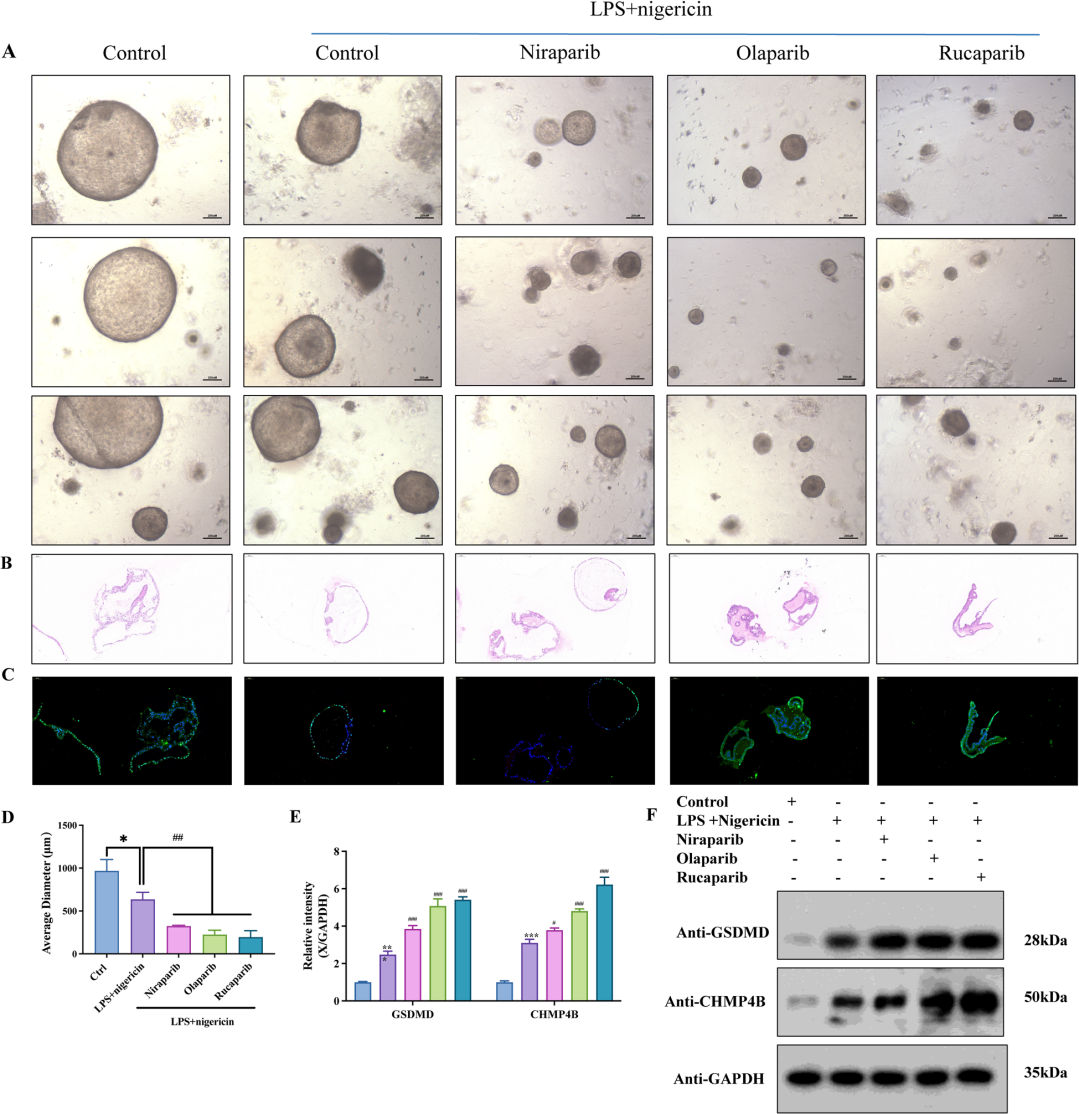
PARP inhibition enhances GSDMD-dependent pyroptosis in endometrial cancer organoids. **(A)** Patient-derived organoids (PDOs) were established from fresh tumor tissues via enzymatic digestion and 3D culture. PDOs were treated with PARP inhibitors—Niraparib (50 nM), Olaparib (200 nM), or Rucaparib (1 μM)—for 24 h, followed by stimulation with LPS (50 ng/mL, 4 h) and Nigericin (10 μM, 30 min). **(B)** Hematoxylin and eosin (H&E) staining of organoids across different treatment groups. **(C)** TUNEL staining revealed enhanced pyroptosis in PARPi-treated groups (scale bar: 100 μm). Organoid diameters were quantified; *P < 0.05, #P < 0.01. **(D)** Organoid size under inverted fluorescence microscopy showed significant reduction in PARPi-treated groups compared to controls. Quantification of average organoid diameter showed a statistically significant decrease following Niraparib, Olaparib, and Rucaparib treatments (*P<0.05, #P<0.01; scale bar: 200 μm). **(E)** Western blot grayscale analysis across treatment groups. **(F)** Western blot results showing upregulation of GSDMD and CHMP4B following PARPi treatment (n = 3, mean ± SD).

Compared to vehicle-treated controls, all three PARPi significantly reduced organoid size post-pyroptotic induction (**Figures 7A, D**, p<0.01). TUNEL and hematoxylin-eosin (HE) staining revealed significant disruption of membrane integrity in endometrial cancer organoids following PARP inhibitor (PARPi) treatment (**Figures 7B, C**). Western blot analysis further revealed a marked upregulation of GSDMD and CHMP4B upon PARPi treatment (**Figures 7D, E**). These findings suggest that PARPi enhances GSDMD-mediated pyroptosis in endometrial cancer PDOs.

### PARPi targets TSG101 to impair CHMP4B membrane remodeling

**Figure 8:**
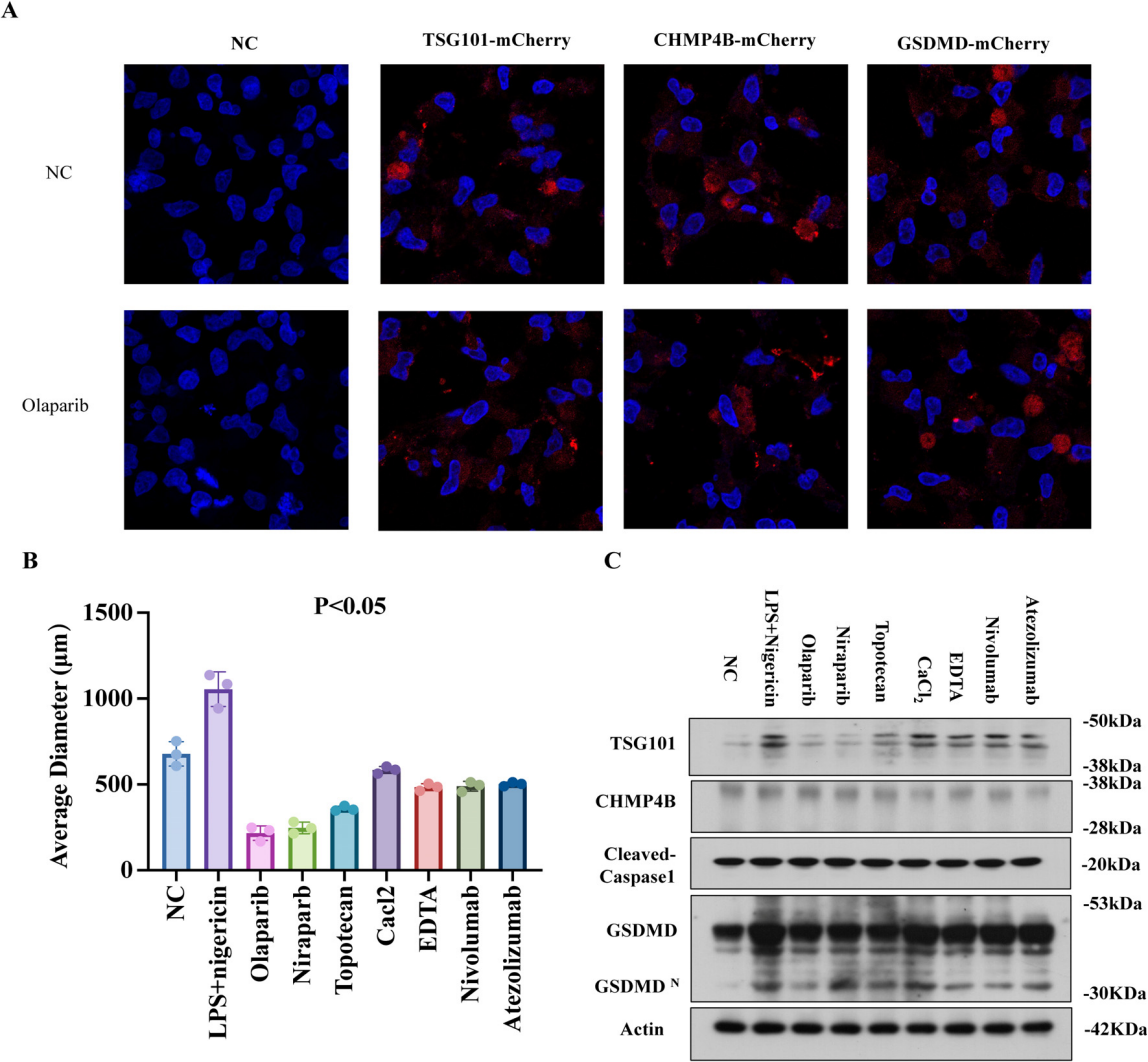
PARPi targets TSG101 to impair CHMP4B membrane remodeling. **(A)** LPS+Nigericin (30 min) induced GSDMDNT-mediated pyroptosis in EC organoids. mCherry-tagged TSG101, CHMP4B, and GSDMD were transfected into organoids. Time-lapse confocal microscopy (10:00 min post-membrane damage) showed PARPi (Olaparib, 24 h) reduced TSG101/CHMP4B membrane localization while enhancing GSDMD clustering (×600). **(B)** Organoid size reduction under PARPi (Olaparib/Niraparib), TSG101 inhibitor (Topotecan HCl), Ca2+ modulators (EDTA/CaCl2), and immune checkpoint inhibitors (Nivolumab/Atezolizumab) without LPS/Nigericin. **(C)** WB demonstrated TSG101/CHMP4B downregulation and Cleaved Caspase-1/GSDMD-N upregulation across treatments. PARPi groups exhibited the most pronounced GSDMD elevation and CHMP4B suppression (mean ± SD).

Time-lapse confocal microscopy of mCherry-tagged TSG101, CHMP4B, and GSDMD in EC organoids revealed that olaparib (24 h) reduced TSG101/CHMP4B membrane localization while enhancing GSDMD clustering at damaged membranes (**Figure 8A**). Without pyroptosis inducers, PARPi (olaparib/niraparib), TSG101 inhibitor (topotecan), Ca²^+^ modulators (EDTA/CaCl_2_), and immune checkpoint inhibitors (nivolumab/atezolizumab) all suppressed organoid growth (**Figure 8B**). WB analysis demonstrated consistent TSG101/CHMP4B downregulation and cleaved caspase-1/GSDMD-N upregulation across treatments, with PARPi showing the strongest CHMP4B suppression and GSDMD elevation (**Figure 8C**, mean ± SD). These findings suggest PARPi impedes TSG101-ESCRT-mediated CHMP4B membrane repair, exacerbating pyroptosis-driven tumor suppression.

## Discussion

In this study, we conducted a comprehensive analysis of the role of the GSDMD gene in cancer prognosis, immunity, and drug therapy from both a pan-cancer and single-cell perspective. Our findings suggest that GSDMD holds promise as a predictive biomarker for cancer immunotherapy.

GSDMD, initially identified as a pyroptosis executor in monocytes (27), regulates immune responses through myeloid cell activation (macrophages/dendritic cells/monocytes) (28), driving both protective immunity and tissue damage (29), while its deficiency impairs neutrophil IL-1β release and myeloid recruitment (30). It mediates pathogen containment by blocking Candida albicanse scape from macrophages (31), amplifies anti-tumor immunity via inflammasome-induced neutrophil pyroptosis (32), and facilitates neutrophil extracellular trap formation through pyroptotic macrophage-derived GSDMD-N+ mitochondrial transfer (33). Pathologically, it contributes to crush syndrome-induced kidney injury via myoglobin-triggered M1 macrophage polarization through the RIG-I/Caspase-1/GSDMD axis (33), demonstrating its multifaceted roles in immunity and disease.

GSDMD oligomerizes to form membrane pores, triggering cell swelling and rupture. Emerging evidence reveals its dual regulatory roles in tumor biology: High expression promotes tumor invasion and predicts poor prognosis in ACC and GBM (consistent with TCGA data) (34, 35) while cytoplasmic localization correlates with favorable immune microenvironment. Conversely, nuclear localization drives metastasis (36). Intriguingly, GSDMD exhibits opposing effects in CRC and SKCM—serving as a positive prognostic marker in CRC (20) while suppressing proliferation and metastasis in melanoma (37). This functional heterogeneity is governed by multiple factors including subcellular localization, tumor microenvironment, and metastasis status, highlighting its context-dependent complexity in cancer progression.

The dual prognostic roles of GSDMD in ACC/LGG versus PRAD/MESO may stem from its context-dependent functional duality. First, GSDMD-mediated pyroptosis exhibits tumor-suppressive effects by triggering immunogenic cell death, yet excessive inflammation from sustained pyroptosis may conversely foster pro-tumorigenic microenvironments. Second, subcellular localization differences could critically modulate its activity—nuclear translocation of GSDMD has been shown to inhibit some malignant phenotypes of colorectal cancer. Third, tumor-type-specific immune landscapes likely contribute: PRAD and MESO typically exhibit higher immunocyte infiltration, where GSDMD-driven pyroptosis may synergize with anti-tumor immunity, whereas ACC/LGG’s immunosuppressive niches might convert pyroptotic debris into pro-metastatic signals. Finally, crosstalk with oncogenic pathways could override GSDMD’s tumor-suppressive potential.

The negative association between TMB and GSDMD may reflect two interconnected biological processes. First, tumors with high TMB often exhibit enhanced immune evasion mechanism (6), which could suppress pyroptosis by impairing inflammasome activation—a prerequisite for GSDMD cleavage. Second, genomic instability caused by high TMB may select for cancer cells that downregulate pyroptotic pathways as a survival strategy, favoring alternative cell death modalities with lesser immunogenic potential (7). Notably, thymomas frequently harbor mutations in epigenetic regulators (8), which may epigenetically silence GSDMD while promoting immune tolerance through thymic epithelial cell dysfunction.

GSDMD expression is correlated with MSI scores in COAD, UCS, and CESC, as well as with key MMR genes, suggesting a significant link between GSDMD expression and immunotherapy response, particularly in UCEC. GSDMD-dependent pathways induce cell pyroptosis, which is accompanied by necrosis and immunogenic cell death, thereby effectively initiating *in situ* immunity (4). *In situ* immunity can enhance the effectiveness of immunotherapies, such as checkpoint inhibitors, by increasing the immunogenicity of tumors (38), hereby improving response rates and treatment outcomes for patients undergoing immunotherapy. Pyroptosis, induced by the Gasdermin family, is emerging as a key defense mechanism in host defense against pathogens (39). Notably, researchers have developed an extracellular vesicle (EV)-based delivery system for GSDMD-N mRNA, which induces pyroptosis and subsequently enhances the effectiveness of immunotherapy (40).

At the single-cell level, GSDMD knockout influences the composition and state progression of immune cells, particularly by increasing T cell proportions while decreasing B cells and macrophages. Despite genetic ablation of Gsdmd, we observed elevated Gsdmd mRNA expression in T cells and dendritic cells in the knockout mice. This paradoxical upregulation may reflect a compensatory feedback mechanism triggered by impaired pyroptosis, leading to increased transcriptional activity. Alternatively, shifts in immune cell composition or inflammatory signals within the tumor microenvironment may contribute (41). Further investigation is warranted to clarify the regulatory dynamics of Gsdmd expression across different immune compartments. Besides, it is important to note that our single-cell RNA-seq analysis was based on a murine tumor model (GSE198550), whereas the bulk transcriptomic and clinical data were derived from human cancers. While interspecies comparisons can offer valuable insights into conserved immune mechanisms, they also carry inherent limitations due to differences in immune cell composition, gene regulation, and tumor microenvironmental context. Future studies leveraging human single-cell datasets are needed to validate our observations and further elucidate the cellular specificity of GSDMD expression.

GSDMD-immune interplay was investigated through multi-platform analysis: Single-cell data (TISCH) revealed high expression in monocytes, macrophages, and malignant cells, while TCGA pan-cancer analysis highlighted its significant association with M2 macrophages, CD8+ T cells, and monocytes (particularly in LAML, LGG). Tumor xenograft models further linked GSDMD to B cell, T cell and macrophage dynamics. Discrepancies across methods may arise from: 1) Species-specific differences. The single-cell RNA-seq data (GSE198550) were derived from a mouse model, whereas the pancancer analysis was based on human datasets. Potential interspecies differences in gene expression and immune cell function may influence the interpretation and translational relevance of the findings. 2) Technical variations in data processing (normalization, statistical pipelines), and 3) Biological heterogeneity (tumor microenvironment, stage).

Future studies should focus on addressing the following aspects: While our study provides initial evidence for GSDMD’s role in immune modulation, the precise molecular mechanisms underlying its effects on immune cell activation and pyroptosis remain unclear. Further mechanistic studies are necessary to elucidate how GSDMD interacts with immune checkpoint pathways, inflammasomes, and the tumor microenvironment. Besides, to strengthen the clinical relevance of GSDMD as a therapeutic target, future work should include the analysis of GSDMD expression in a broader cohort of clinical samples, especially in the context of immunotherapy. Furthermore, preclinical studies using GSDMD-modified animal models should be employed to explore the therapeutic potential of targeting GSDMD. Combining GSDMD-targeted therapies with current immunotherapies may provide synergistic effects and significantly improve treatment outcomes in patients with GSDMD-high tumors.

## Conclusion

Given its involvement in multiple stages of cancer development and progression, GSDMD represents a promising therapeutic target for cancer treatment. Strategies aimed at modulating GSDMD expression or activity, either alone or in combination with existing therapies, hold potential for improving treatment outcomes and overcoming drug resistance. Moreover, targeting GSDMD-mediated pyroptosis may offer a novel approach to harnessing the immune system for anti-tumor immunity.

## Data Availability

RNA-seq data for 33 tumor types and corresponding normal tissues were obtained from the TCGA database (https://portal.gdc.cancer.gov/, accessed January 6, 2024) and the GTEx database (https://gtexportal.org, accessed January 5, 2024). The single-cell data is sourced from GSE198550 dataset from NCBI (https://www.ncbi.nlm.nih.gov/geo/query/acc.cgi?acc=GSE198550).

## Glossary

GSDMD: Gasdermin D
GTEx: Genotype-Tissue Expression
TCGA: The Cancer Genome Atlas Program
TMB: Tumor Mutation Burden
MSI: Microsatellite Instability
MMR: Mismatch Repair
TME: Tumor microenvironment
ACC: Adrenocortical Carcinoma
BLCA: Bladder Urothelial Carcinoma
BRCA: Breast Invasive Carcinoma
CESC: Cervical Squamous Cell Carcinoma And Endocervical Adenocarcinoma
CHOL: Cholangiocarcinoma
COAD: Colon Adenocarcinoma
DLBC: Lymphoid Neoplasm Diffuse Large B-Cell Lymphoma
ESCA: Esophageal Carcinoma
GBM: Glioblastoma Multiforme
HNSC: Head And Neck Squamous Cell Carcinoma
KICH: Kidney Chromophobe
KIRC: Kidney Renal Clear Cell Carcinoma
KIRP: Kidney Renal Papillary Cell Carcinoma
LAML: arcin Myeloid Leukemia
LGG: Brain Lower Grade Glioma
LIHC: Liver Hepatocellular Carcinoma
LUAD: Lung Adenocarcinoma
LUSC: Lung Squamous Cell Carcinoma
MESO: Mesothelioma
OV: Ovarian Serous Cystadenocarcinoma
PAAD: Pancreatic Adenocarcinoma
PCPG: Pheochromocytoma And Paraganglioma
PRAD: Prostate Adenocarcinoma
READ: Rectum Adenocarcinoma
SARC: Sarcoma
SKCM: Skin Cutaneous Melanoma
STAD: Stomach Adenocarcinoma
TGCT: Testicular Germ Cell Tumors
THCA: Thyroid Carcinoma
THYM: Thymoma
UCEC: Uterine Corpus Endometrial Carcinoma
UCS: Uterine Carcinosarcoma
UVM: cinosa Melanoma

## References

[1] Sharma P Hu-Lieskovan S Wargo JA Ribas A. Primary, adaptive, and acquired resistance to cancer immunotherapy. Cell. (2017) 168:707–23. doi: 10.1016/j.cell.2017.01.017 PMC5391692 28187290

[2] Konieczkowski DJ Johannessen CM Garraway LA. A convergence-based framework for cancer drug resistance. Cancer Cell. (2018) 33:801–15. doi: 10.1016/j.ccell.2018.03.025 PMC5957297 29763622

[3] Batis N Brooks JM Payne K Sharma N Nankivell P Mehanna H. Lack of predictive tools for conventional and targeted cancer therapy: Barriers to biomarker development and clinical translation. Advanced Drug Delivery Rev. (2021) 176:113854. doi: 10.1016/j.addr.2021.113854 PMC8448142 34192550

[4] Ding B Chen H Tan J Meng Q Zheng P Ma P. ZIF-8 nanoparticles evoke pyroptosis for high-efficiency cancer immunotherapy. Angew Chem Int Ed Engl. (2023) 62:e202215307. doi: 10.1002/anie.202215307 36629270

[5] Wei X Xie F Zhou X Wu Y Yan H Liu T. Role of pyroptosis in inflammation and cancer. Cell Mol Immunol. (2022) 19:971–92. doi: 10.1038/s41423-022-00905-x PMC9376585 35970871

[6] Qiu S Hu Y Dong S. Pan-cancer analysis reveals the expression, genetic alteration and prognosis of pyroptosis key gene GSDMD. Int Immunopharmacol. (2021) 101:108270. doi: 10.1016/j.intimp.2021.108270 34700129

[7] Yang Y Liu PY Bao W Chen SJ Wu FS Zhu PY. Hydrogen inhibits endometrial cancer growth via a ROS/NLRP3/caspase-1/GSDMD-mediated pyroptotic pathway. BMC Cancer. (2020) 20:28. doi: 10.1186/s12885-019-6491-6 31924176 PMC6954594

[8] Zheng Y Yuan D Zhang F Tang R. A systematic pan-cancer analysis of the gasdermin (GSDM) family of genes and their correlation with prognosis, the tumor microenvironment, and drug sensitivity. Front Genet. (2022) 13:926796. doi: 10.3389/fgene.2022.926796 36003332 PMC9393220

[9] Yan H Luo B Wu X Guan F Yu X Zhao L. Cisplatin induces pyroptosis via activation of MEG3/NLRP3/caspase-1/GSDMD pathway in triple-negative breast cancer. Int J Biol Sci. (2021) 17:2606–21. doi: 10.7150/ijbs.60292 PMC8315016 34326697

[10] Li T Fu J Zeng Z Cohen D Li J Chen Q. TIMER2.0 for analysis of tumor-infiltrating immune cells. Nucleic Acids Res. (2020) 48:W509–w514. doi: 10.1093/nar/gkaa407 32442275 PMC7319575

[11] Steuer CE Ramalingam SS. Tumor mutation burden: leading immunotherapy to the era of precision medicine? J Clin Oncol. (2018) 36:631–2. doi: 10.1200/jco.2017.76.8770 29337637

[12] Choucair K Morand S Stanbery L Edelman G Dworkin L Nemunaitis J. TMB: a promising immune-response biomarker, and potential spearhead in advancing targeted therapy trials. Cancer Gene Ther. (2020) 27:841–53. doi: 10.1038/s41417-020-0174-y 32341410

[13] van Velzen MJM Derks S van Grieken NCT Haj Mohammad N van Laarhoven HWM. MSI as a predictive factor for treatment outcome of gastroesophageal adenocarcinoma. Cancer Treat Rev. (2020) 86:102024. doi: 10.1016/j.ctrv.2020.102024 32388292

[14] Baretti M Le DT. DNA mismatch repair in cancer. Pharmacol Ther. (2018) 189:45–62. doi: 10.1016/j.pharmthera.2018.04.004 29669262

[15] Shen W Song Z Zhong X Huang M Shen D Gao P. Sangerbox: A comprehensive, interaction-friendly clinical bioinformatics analysis platform. iMeta. (2022) 1:e36. doi: 10.1002/imt2.v1.3 38868713 PMC10989974

[16] Huang H Tang Y Li P Ye X Chen W Xie H. Significance of TP53 and immune-related genes to prostate cancer. Transl Androl Urol. (2021) 10:1754–68. doi: 10.21037/tau-21-179 PMC8100849 33968663

[17] Wang H You S Fang M Fang Q. Recognition of immune microenvironment landscape and immune-related prognostic genes in breast cancer. BioMed Res Int. (2020) 2020:3909416. doi: 10.1155/2020/3909416 33274208 PMC7683123

[18] Chen Z Luo Z Zhang D Li H Liu X Zhu K. TIGER: A web portal of tumor immunotherapy gene expression resource. Genomics Proteomics Bioinf. (2023) 21:337–48. doi: 10.1016/j.gpb.2022.08.004 PMC10626175 36049666

[19] Jiang Y Yang Y Hu Y Yang R Huang J Liu Y. Gasdermin D restricts anti-tumor immunity during PD-L1 checkpoint blockade. Cell Rep. (2022) 41:111553. doi: 10.1016/j.celrep.2022.111553 36288704

[20] Hu C Li T Xu Y Zhang X Li F Bai J. CellMarker 2.0: an updated database of manually curated cell markers in human/mouse and web tools based on scRNA-seq data. Nucleic Acids Res. (2023) 51:D870–6. doi: 10.1093/nar/gkac947 PMC9825416 36300619

[21] Jin S Guerrero-Juarez CF Zhang L Chang I Ramos R Kuan CH. Inference and analysis of cell-cell communication using CellChat. Nat Commun. (2021) 12:1088. doi: 10.1038/s41467-021-21246-9 33597522 PMC7889871

[22] Zhao J Chen AX Gartrell RD Silverman AM Aparicio L Chu T. Immune and genomic correlates of response to anti-PD-1 immunotherapy in glioblastoma. Nat Med. (2019) 25:462–9. doi: 10.1038/s41591-019-0349-y PMC6810613 30742119

[23] Riaz N Havel JJ Makarov V Desrichard A Urba WJ Sims JS. Tumor and microenvironment evolution during immunotherapy with nivolumab. Cell. (2017) 171:934–949.e916. doi: 10.1016/j.cell.2017.09.028 29033130 PMC5685550

[24] Nathanson T Ahuja A Rubinsteyn A Aksoy BA Hellmann MD Miao D. Somatic mutations and neoepitope homology in melanomas treated with CTLA-4 blockade. Cancer Immunol Res. (2017) 5:84–91. doi: 10.1158/2326-6066.CIR-16-0019 27956380 PMC5253347

[25] Jung H Kim HS Kim JY Sun JM Ahn JS Ahn MJ. DNA methylation loss promotes immune evasion of tumours with high mutation and copy number load. Nat Commun. (2019) 10:4278. doi: 10.1038/s41467-019-12159-9 31537801 PMC6753140

[26] Yang Y Chen HL Wu SF Bao W. CHMP4B and VSP4A reverse GSDMD-mediated pyroptosis by cell membrane remodeling in endometrial carcinoma. Biochim Biophys Acta Gen Subj. (2024) 1868:130497. doi: 10.1016/j.bbagen.2023.130497 37931722

[27] Shi J Gao W Shao F. Pyroptosis: gasdermin-mediated programmed necrotic cell death. Trends Biochem Sci. (2017) 42:245–54. doi: 10.1016/j.tibs.2016.10.004 27932073

[28] Li Y Jiang Q. Uncoupled pyroptosis and IL-1β secretion downstream of inflammasome signaling. Front Immunol. (2023) 14:1128358. doi: 10.3389/fimmu.2023.1128358 37090724 PMC10117957

[29] Zhang H Zeng L Xie M Liu J Zhou B Wu R. TMEM173 drives lethal coagulation in sepsis. Cell Host Microbe. (2020) 27:556–570.e556. doi: 10.1016/j.chom.2020.02.004 32142632 PMC7316085

[30] Jiang K Tu Z Chen K Xu Y Chen F Xu S. Gasdermin D inhibition confers antineutrophil-mediated cardioprotection in acute myocardial infarction. J Clin Invest. (2022) 132:e151268. doi: 10.1172/JCI151268 34752417 PMC8718151

[31] Ding X Kambara H Guo R Kanneganti A Acosta-Zaldívar M Li J. Inflammasome-mediated GSDMD activation facilitates escape of Candida albicans from macrophages. Nat Commun. (2021) 12:6699. doi: 10.1038/s41467-021-27034-9 34795266 PMC8602704

[32] Chauhan D Demon D Vande Walle L Paerewijck O Zecchin A Bosseler L. GSDMD drives canonical inflammasome-induced neutrophil pyroptosis and is dispensable for NETosis. EMBO Rep. (2022) 23:e54277. doi: 10.15252/embr.202154277 35899491 PMC9535806

[33] Li N Chen J Geng C Wang X Wang Y Sun N. Myoglobin promotes macrophage polarization to M1 type and pyroptosis via the RIG-I/Caspase1/GSDMD signaling pathway in CS-AKI. Cell Death Discov. (2022) 8:90. doi: 10.1038/s41420-022-00894-w 35228524 PMC8885737

[34] Shen X Zhang Q He Z Xiao S Li H Huang Z. Overexpression of gasdermin D promotes invasion of adenoid cystic carcinoma. Int J Clin Exp Pathol. (2020) 13:1802–11. PMC7414463 32782708

[35] Liu J Gao L Zhu X Geng R Tao X Xu H. Gasdermin D is a novel prognostic biomarker and relates to TMZ response in glioblastoma. Cancers (Basel). (2021) 13:5620. doi: 10.3390/cancers13225620 34830775 PMC8616249

[36] Wang J Kang Y Li Y Sun L Zhang J Qian S. Gasdermin D in different subcellular locations predicts diverse progression, immune microenvironment and prognosis in colorectal cancer. J Inflammation Res. (2021) 14:6223–35. doi: 10.2147/JIR.S338584 PMC8630373 34858044

[37] Zhao S Zhu Y Liu H He X Xie J. System analysis based on the pyroptosis-related genes identifes GSDMD as a novel therapy target for skin cutaneous melanoma. J Transl Med. (2023) 21:801. doi: 10.1186/s12967-023-04513-9 37950289 PMC10636830

[38] Li Z Lai X Fu S Ren L Cai H Zhang H. Immunogenic cell death activates the tumor immune microenvironment to boost the immunotherapy efficiency. Adv Sci (Weinh). (2022) 9:e2201734. doi: 10.1002/advs.202201734 35652198 PMC9353475

[39] Vasudevan SO Behl B Rathinam VA. Pyroptosis-induced inflammation and tissue damage. Semin Immunol. (2023) 69:101781. doi: 10.1016/j.smim.2023.101781 37352727 PMC10598759

[40] Xing Y Zhang F Ji P Wei M Yin C Yang A. Efficient delivery of GSDMD-N mRNA by engineered extracellular vesicles induces pyroptosis for enhanced immunotherapy. Small. (2023) 19:e2204031. doi: 10.1002/smll.202204031 36635060

[41] de Visser KE Joyce JA. The evolving tumor microenvironment: From cancer initiation to metastatic outgrowth. Cancer Cell. (2023) 41:374–403. doi: 10.1016/j.ccell.2023.02.016 36917948

