# Supplementary figures and images for: GSDMD is a novel predictive biomarker for immunotherapy response: in the pan-cancer and single cell landscapes
Source: Front Immunol. 2025 May 26;16:1570901. doi: 10.3389/fimmu.2025.1570901 (PMC12146313; doi:10.3389/fimmu.2025.1570901)

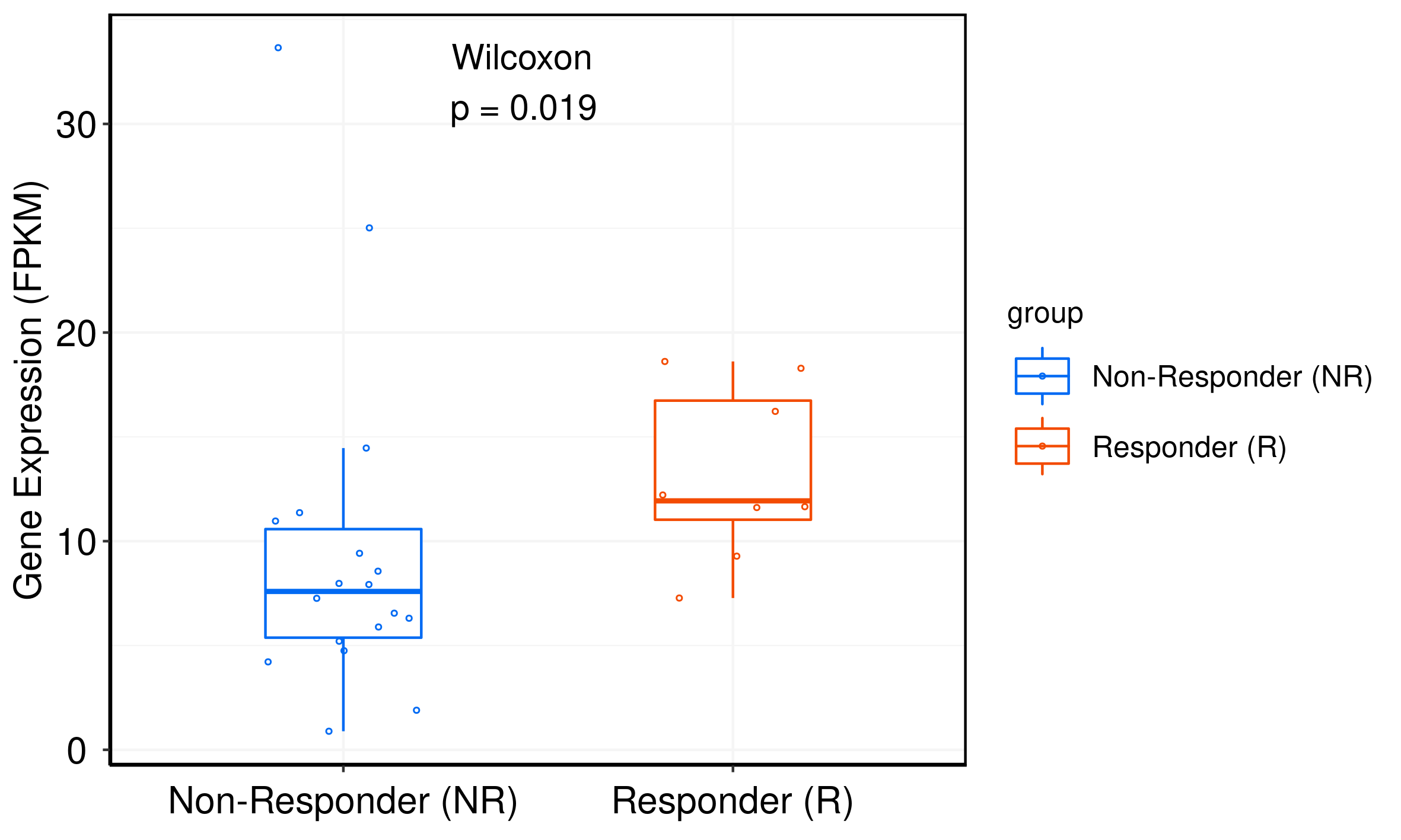

Supplement: Supplementary file 1 [file Image1.png]

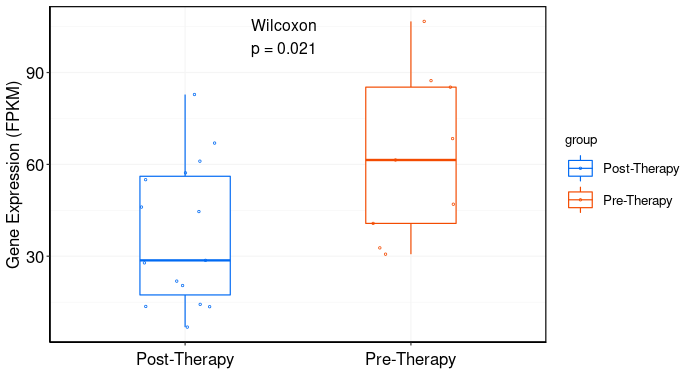

Supplement: Supplementary file 2 [file Image2.png]

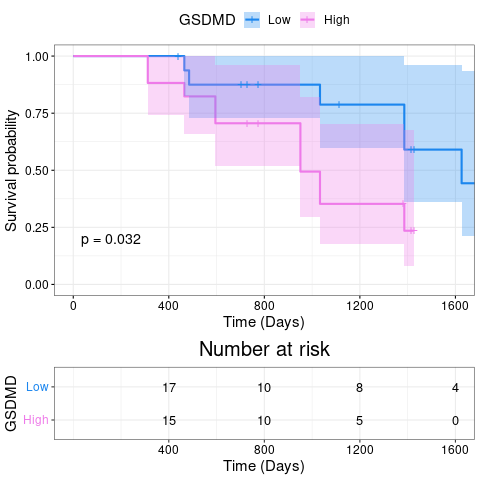

Supplement: Supplementary file 3 [file Image3.png]

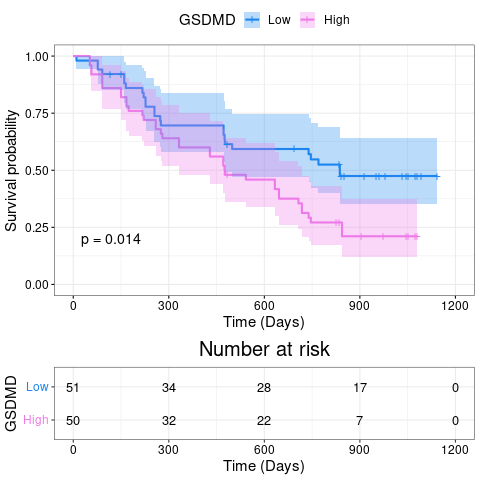

Supplement: Supplementary file 4 [file Image4.png]
